# Hepatic Angiosarcoma: A Case Presentation

**DOI:** 10.7759/cureus.6848

**Published:** 2020-02-02

**Authors:** Muhammad Noor, Alexander Leyva, Stephen J Patacsil, Midhir Patel, Christopher Smith

**Affiliations:** 1 Radiology, AdventHealth Orlando, Orlando, USA

**Keywords:** hepatic angiosarcoma, hepatic tumors

## Abstract

A 71-year-old male who presented with right upper quadrant pain was found to have hepatic angiosarcoma. We briefly review the epidemiology, presentation, imaging findings, and pathological diagnosis of hepatic angiosarcoma.

## Introduction

Hepatic angiosarcoma, although rare, is the third most common primary liver malignancy. We present a case of a 71-year-old-male who presented with right upper quadrant pain and subsequently diagnosed with hepatic angiosarcoma during admission. We briefly discuss the epidemiology, imaging characteristics, and pathological diagnosis of hepatic angiosarcoma. 

## Case presentation

**Figure 1:**
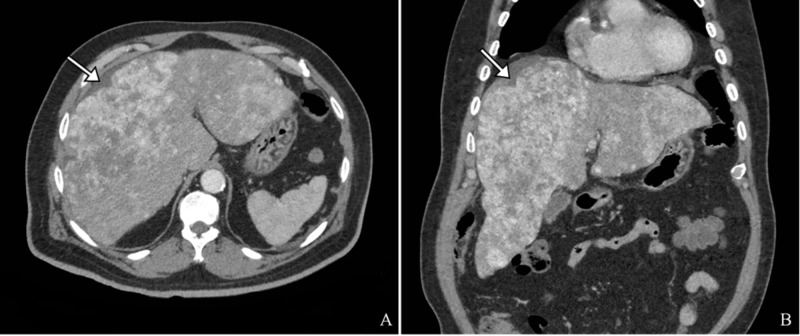
Representative CT images Contrast-enhanced CT images in the axial (A) and coronal (B) planes demonstrate a diffusely enhancing, multifocal, heterogeneous, and infiltrative mass occupying most of the hepatic parenchyma. Mild capsular retraction (arrows) is noted secondary to the mass at the periphery of the liver. CT findings are highly concerning for a primary or metastatic hepatic malignancy.

A 71-year-old male with a remote history of prostate cancer and diabetes presented with a one-week history of gradually worsening right upper quadrant abdominal pain. A contrast-enhanced computed tomography (CT) scan (Figure 1) was obtained in the emergency department, which revealed a large multifocal and diffusely enhancing mass within the liver. Laboratory workup revealed a slightly elevated aspartate aminotransferase, but was otherwise unremarkable. Tumor markers, including carcinoembryonic antigen, alpha fetoprotein, and carbohydrate antigen 19-9, were negative. The patient was subsequently admitted for further evaluation.

**Figure 2:**
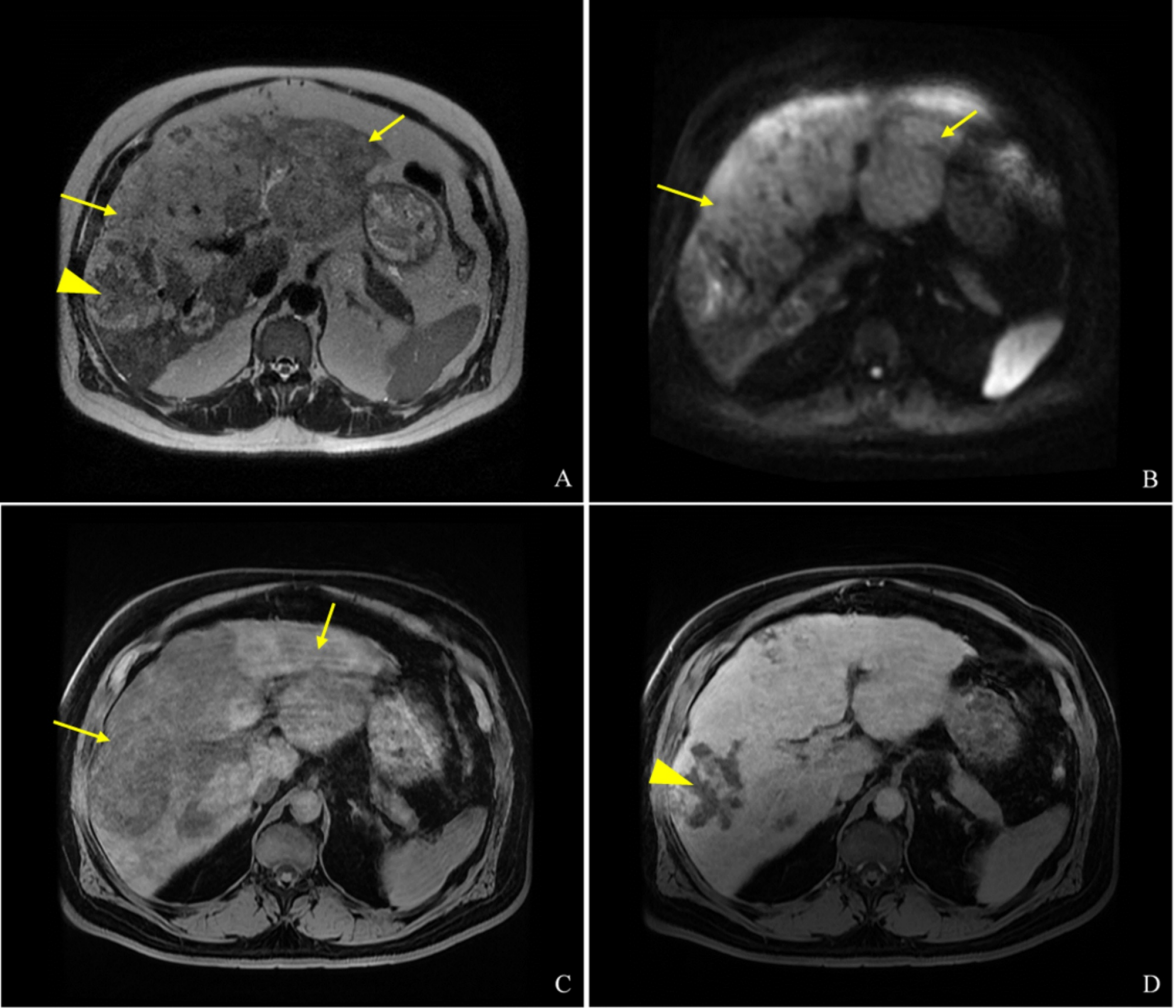
Representative MRI images Multiple axial MR images. (A) T2-weighted image demonstrates a largely T2 hyperintense infiltrative mass occupying the hepatic parenchyma (arrows) with scattered regions of T2 hypointensity likely representing necrosis (arrowhead). (B) The diffusion-weighted MR image demonstrates restricted diffusion throughout the mass-corresponding apparent diffusion coefficient images demonstrate hypointensity confirming diffusion restriction (arrows). (C) Precontrast T1-weighted MR image demonstrates relative hypointense signal in the region of the mass (arrows). (D) Gadolinium contrast-enhanced T1-weighted MR image demonstrates diffuse enhancement with regions of non-enhancement corresponding to the areas of necrosis (arrowhead).

Magnetic resonance (MR) images obtained during admission showed a multifocal, predominantly T2 hyperintense hepatic mass, with heterogeneous enhancement and areas of necrosis (Figure 2). Further imaging workup was negative for signs of extrahepatic metastatic disease.

**Figure 3:**
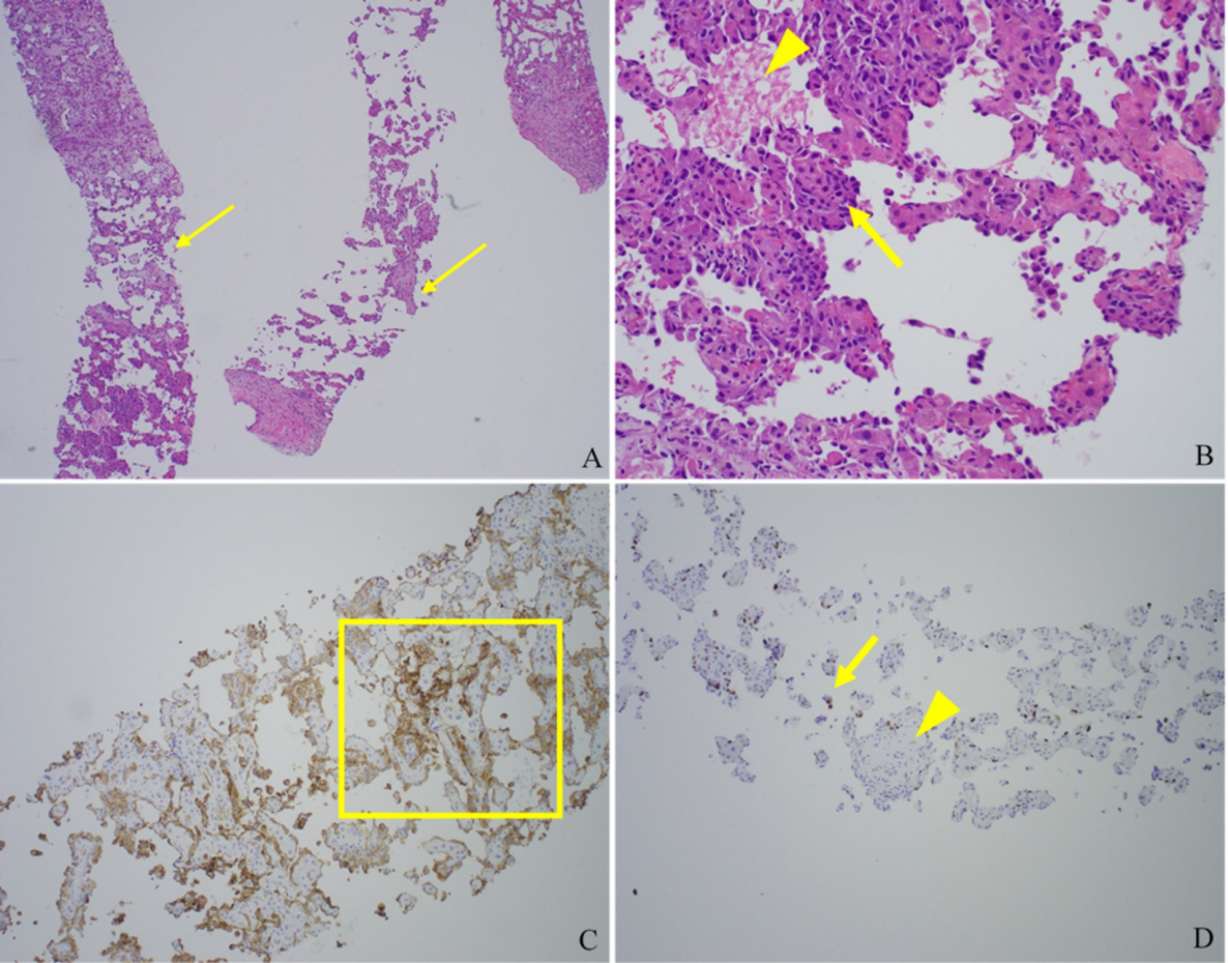
Representative pathology slides from a liver core biopsy (A) Low-power view of the patient’s liver biopsy showing the abnormal architecture of freely anastomosing vascular channels (arrows). (B) Small groups of hepatocytes (arrowhead) surrounded by large abnormal endothelial cells (arrow). (C) CD31 stain highlighting the abnormal endothelial cells (representative area within square). (D) Increased MIB-1 proliferation index noted in the endothelial cells (arrow) and not in the hepatocytes (arrowhead).

Results from a liver core biopsy (Figure 3) demonstrate abnormal hepatic architecture with freely anastomosing vasculature and large, abnormal CD31+ endothelial cells surrounding small groups of hepatocytes-findings consistent with hepatic angiosarcoma.

## Discussion

Hepatic angiosarcoma is the most common primary mesenchymal tumor of the liver and the third most common primary liver malignancy [1]. Hepatic angiosarcoma is a rare malignancy that accounts for less than 2% of all primary liver malignancies and appears more frequently in male patients older than 60 years of age [2]. Patients are typically asymptomatic until later stages of disease when they may present with abdominal pain or hemoptysis due to pulmonary metastasis. Patients who previously received thorium dioxide (Thorotrast) for imaging studies are at increased risk of developing hepatic angiosarcoma [3]. Additionally, occupational exposures to vinyl chloride monomer and arsenic are known to induce its formation.

On multiphasic contrast-enhanced CT and MR exams, hepatic angiosarcoma commonly appears as a multifocal tumor with variable enhancement patterns [2]. It is important to appreciate that hepatic angiosarcoma may share imaging characteristics with more common hepatic tumors. However, some differentiating characteristics do exist. For example, hepatic angiosarcoma with peripheral nodular enhancement can be distinguished from hemangioma by a lack of slow centripetal filling on delayed images [4]. Also, the poorly differentiated cells of hepatic angiosarcoma are less likely to retain hepatobiliary MR contrast agents to the extent of focal nodular hyperplasia [5-7].

On pathological analysis, hepatic angiosarcoma displays infiltrative vascular channels lined by atypical endothelial cells [8-10]. Lesions are typically multifocal, aggressive, and prone to hemorrhage which pose specific challenges against creating an efficacious treatment regimen. Short interval follow-up can be helpful for ambiguous lesions that do not meet criteria for biopsy.

## Conclusions

Hepatic angiosarcoma is a rare entity that is difficult to distinguish from most other hepatic tumors and requires a pathological diagnosis. There are certain imaging characteristics, however, which may necessitate the inclusion of this entity on the differential for a hepatic mass. 
